# Serum bicarbonate concentration and the risk of cardiovascular disease and death in type 2 diabetes: the Fremantle Diabetes Study

**DOI:** 10.1186/s12933-016-0462-x

**Published:** 2016-10-06

**Authors:** S. A. Paul Chubb, Wendy A. Davis, Kirsten E. Peters, Timothy M. E. Davis

**Affiliations:** 1 School of Medicine and Pharmacology, University of Western Australia, Fremantle Hospital, PO Box 480, Fremantle, WA 6959 Australia; 2 PathWest Laboratory Medicine WA, Fiona Stanley Hospital, Murdoch, WA Australia

**Keywords:** Bicarbonate, Coronary artery disease, Type 2 diabetes, Mortality

## Abstract

**Background:**

Serum bicarbonate is associated with mortality, heart failure (HF) and progression of renal failure in studies of healthy people and patients with chronic kidney disease, but the significance of these observations in unselected patients with diabetes in the general population is unknown. The aim of this study was to determine whether serum bicarbonate was associated with mortality and cardiovascular disease risk in type 2 diabetes.

**Methods:**

Baseline serum bicarbonate was available for 1283 well-characterized community-based patients (mean ± SD age 64.1 ± 11.3 years, 48.7 % males) from the longitudinal observational Fremantle Diabetes Study followed for a mean of 12 years. Associations between serum bicarbonate and mortality, coronary heart disease (CHD) and incident HF were analysed using Cox proportional hazards regression.

**Results:**

Serum bicarbonate was independently and negatively associated with incident CHD. For each 1 mmol/L increase in bicarbonate, the hazard ratio for CHD was 0.95 (95 % confidence interval 0.92–0.99) after adjustment for age as time scale, age at baseline, sex, English fluency, diabetes duration, log_e_(serum triglycerides), log_e_(urinary albumin: creatinine ratio), peripheral sensory neuropathy and peripheral arterial disease. There were no independent associations between serum bicarbonate and all-cause mortality [0.98 (0.95–1.004)] or incident HF [0.99 (0.95–1.03)].

**Conclusions:**

Serum bicarbonate was a significant independent predictor of incident CHD but not death or HF in community-based patients with type 2 diabetes. This supports intervention trials of bicarbonate replacement in type 2 patients at risk of CHD and who have a low serum bicarbonate concentration.

**Electronic supplementary material:**

The online version of this article (doi:10.1186/s12933-016-0462-x) contains supplementary material, which is available to authorized users.

## Background

Serum bicarbonate is typically included in routine biochemical tests of renal function and is therefore likely to be measured regularly as part of the long term management of patients with type 2 diabetes. Clinically significant abnormalities of bicarbonate homoeostasis are rare in this setting. Indeed, guidelines recommend its measurement as an index of acid–base balance only when renal disease has developed [1]. However, despite these considerations, some population-based studies and data from cohorts of patients with chronic kidney disease (CKD) have suggested a possible role for serum bicarbonate as an independent risk marker of mortality [2–9] and heart failure (HF) [2, 10] in addition to progressive renal disease [2, 3, 11, 12]. Just one of these studies included only patients with diabetes and all had established CKD [10]. This and other less selective studies of patients with CKD [2, 4, 5] have suggested that an association between serum bicarbonate and mortality may be lost in diabetes.

There has been no investigation of the relationship of serum bicarbonate and key endpoints including death in unselected community-based patients with diabetes with renal function ranging from normal to end-stage renal disease. Such a study could provide evidence of the utility of a routinely-measured serum bicarbonate as a prognostic indicator that could prompt modification of therapy and thus potentially reduce morbidity and death. We have, therefore, assessed the associations between serum bicarbonate and all-cause mortality, coronary heart disease (CHD) and HF in well characterized representative community-dwelling patients with type 2 diabetes. We hypothesized that serum bicarbonate would have significant independent associations with mortality, CHD and HF in this cohort.

## Methods

### Patients, epidemiological setting and approvals

The Fremantle Diabetes Study (FDS) is a prospective observational study of diabetes in a zip code-defined urban population of approximately 120,000 people living in and around the port city of Fremantle in Western Australia (WA) [13]. The residents of the FDS catchment area appear representative of the general Australian population. Socio-economic data relating to income, employment, housing, transportation and a range of other variables show an average index of relative socio-economic advantage and disadvantage [14] of 1033 with a range by zip code of 977-1113, figures similar to the national mean ± SD which are set at 1000 ± 100.

All patients with physician diagnosed diabetes living in or near the port city of Fremantle, Western Australia were eligible to take part in the FDS. Of 2258 patients identified between 1993 and 1996, 1426 (63 %) were recruited of whom 1296 (91 %) had type 2 diabetes based on demographic, anthropometric, clinical and laboratory features. Eligible patients who were not recruited were a mean of 1.4 years older than participants, but their sex distribution, diabetes type, and blood glucose-lowering treatment were similar [13]. The FDS was approved by the Fremantle Hospital Human Research Ethics Committee and all patients gave written informed consent.

### Clinical methods

Participants had comprehensive face-to-face assessments at baseline and annually for up to 5 years thereafter [13]. At each visit, demographic and clinical information including details of other illnesses, were documented, physical examinations and associated investigations were carried out, and fasting samples for biochemical tests were obtained. Micro- and macrovascular complications of diabetes at study entry were identified using standard criteria [13, 15], including peripheral sensory neuropathy (a score of >2/8 on the clinical portion of the Michigan Neuropathy Screening Instrument [16]), retinopathy (any grade detected by direct/indirect ophthalmoscopy and/or ophthalmologist assessment), nephropathy (first-morning urinary albumin: creatinine ratio >3.0 mg/mmol), renal impairment [by estimated glomerular filtration rate (eGFR) determined using the chronic kidney disease epidemiology collaboration (CKD-EPI) equation [17]], CHD (self-reported history of myocardial infarction, angina and/or revascularization, or prior hospitalizations for these events), cerebrovascular disease (self-reported stroke/transient ischemic attack or prior hospitalizations for these events), and peripheral arterial disease (ankle:brachial index ≤0.90 on either leg or diabetes-related amputation). The Charlson comorbidity index (CCI) was calculated as a measure of chronic disease morbidity after excluding conditions related to diabetes [18].

Endpoints were ascertained from the WA Data Linkage System (WADLS). This includes the Hospital Morbidity Data System which documents all hospitalizations (public or private) in WA since 1970 and all WA death registrations [19]. The WADLS was linked to the FDS database from the beginning of the study until end-December 2012 for deaths and, to ensure complete ascertainment of hospitalizations, to end-June 2012 for incident CHD and HF. Incident CHD was defined as first ever hospitalization for/with CHD or death due to cardiac causes or sudden death between study entry in 1993–96 and end-June 2012. Incident HF was defined as first ever hospitalization for/with HF or death from/with HF between study entry in 1993–96 and end-June 2012.

### Biochemical assays

Morning fasting venous blood samples were collected from each patient, centrifuged promptly and sent for prompt analysis of standard biochemical parameters in a single nationally accredited laboratory. Spot first-morning urine samples were also collected. Serum bicarbonate was analysed by the phospho*enol*pyruvate carboxylase method with between-run imprecision (expressed as coefficient of variation) of 1.9 % at 22.7 mmol/L and 1.1 % at 28.8 mmol/L. Serum potassium, chloride, creatinine, glucose, cholesterol, triglycerides and HDL-cholesterol, as well as urine albumin and creatinine, were measured by standard methods on a Hitachi 911 analyser (Roche Diagnostics Australia, Castle Hill, NSW, Australia). Glycated hemoglobin was estimated by cation-exchange high-performance liquid chromatography using a Mono S HR 5/5 column (Amersham Biosciences, Castle Hill, NSW, Australia) [20].

### Data analysis

The computer packages IBM SPSS Statistics 22 (IBM Corporation, Armonk, NY, USA) and StataIC 13 (College Station, TX: StataCorp LP) were used for statistical analysis. Data are presented as proportions, mean ± SD, geometric mean (SD range), or, in the case of variables which did not conform to a normal or log-normal distribution, median and [inter-quartile range]. For independent samples, two-way comparisons for proportions were by Fisher’s exact test, for normally distributed variables by Student’s *t* test, and for non-normally distributed variables by Mann–Whitney *U*-test. Multiple comparisons for proportions were by Fisher’s exact test or Chi squared test, for normally distributed variables by ANOVA, and for non-normally distributed variables by Kruskal–Wallis test. For statistically significant trends, pairwise comparisons were adjusted for multiple comparisons using the Bonferroni correction.

Multiple linear regression using stepwise entry was performed (*P* < 0.05 for entry, *P* > 0.10 for removal) to identify independent associates of serum bicarbonate at baseline. Clinically or biologically plausible variables with *P* < 0.20 in bivariate analyses across quintiles of bicarbonate were considered for entry into the model. For analysis of predictors of all-cause death, incident CHD and incident HF in FDS1 type 2 participants, bivariate baseline associates of all-cause death, incident CHD and incident HF were first determined (see Additional file 1). Cox proportional hazards modelling, with age as the time scale and left truncation at study entry, employed due to the presence of covariates strongly associated with age [21], was used to determine independent predictors of all-cause death, incident CHD and incident HF, excluding serum bicarbonate, using interactive forward conditional modelling (*P* < 0.05 for entry, *P* > 0.10 for removal) with all clinically plausible variables at *P* < 0.20 in bivariate analyses considered for entry. After adjustment for the most parsimonious model (see Additional file 2), serum bicarbonate as a continuous variable or as quintiles was entered. Five supplementary analyses were undertaken for each outcome to adjust for a range of variables from none to a fully adjusted model (see Additional file 3). Restricted cubic spline modelling with three and four knots was undertaken to confirm the shape of the relationship between serum bicarbonate and incident CHD risk with a reference set at 28 mmol/L, the lowest risk serum bicarbonate quintile.

## Results

### Baseline characteristics

Serum bicarbonate was available for 1283 of the 1296 patients (99.0 %; mean ± SD age 64.1 ± 11.3 years, 48.7 % males) with type 2 diabetes in the FDS cohort. Their mean ± SD serum bicarbonate was 27 ± 3 mmol/L. The demographic, clinical and biochemical characteristics of patients in each quintile of serum bicarbonate are summarized in Table 1. Patients in the upper quintiles were more likely to be older and male, to have been diagnosed with diabetes at an older age, to be taking anti-hypertensive medications and/or diuretics, and to have a history of peripheral vascular disease or CHD. Patients in the lower quintiles were likely to have higher serum cholesterol and triglycerides, and a higher body mass index. Patients with eGFR <45 mL/min/1.73 m^2^ were also more likely to have a serum bicarbonate in the lowest quintile. There was a graded inverse association between serum bicarbonate quintile and serum chloride concentration. In multivariate analysis, a higher serum bicarbonate was significantly associated with male sex, age at diabetes diagnosis, insulin treatment, diuretic therapy and total serum cholesterol, and negatively with being an ex-smoker, log_e_(serum triglycerides), serum chloride, CHD and CCI (see Table 2).

**Table 1:** Baseline characteristics of patients with type 2 diabetes in the Fremantle Diabetes Study categorized by quintile of serum bicarbonate

	Serum bicarbonate quintile (mmol/L)					
	Q1 ≤23	Q2 24–25	Q3 26–27	Q4 28	Q5 ≥29	*P* value
N (%)	183 (14.3)	219 (17.1)	341 (26.6)	178 (13.9)	362 (28.2)	
Age (years)	61.9 ± 12.4	62.6 ± 12.8	63.2 ± 10.3	64.3 ± 11.0	66.8 ± 10.1***^,†††, ‡‡‡^	<0.001
Male (%)	43.7	38.8	46.9	57.3^†††^	54.7^†††^	<0.001
Ethnic background (%)						0.22
Anglo-Celt	65.6	62.6	62.2	60.1	58.8	
Southern European	11.5	16.0	19.4	20.8	18.5	
Other European	10.4	7.3	6.2	10.1	9.4	
Asian	1.1	4.1	3.2	3.4	4.4	
Aboriginal	3.3	1.4	1.2	1.7	0.8	
Mixed/other	8.2	8.7	7.9	3.9	8.0	
Not fluent in English (%)	12.0	12.8	17.6	18.5	14.7	0.24
Educated beyond primary school (%)	77.3	73.8	75.7	70.5	72.8	0.55
Currently married/de facto relationship (%)	62.8	61.0	65.4	68.5	69.0	0.27
Alcohol consumption (standard drinks/day)	0 [0–0.8]	0 [0–0.3]	0 [0–0.8]	0 [0.08]	0 [0–0.8]	0.50
Smoking status (%)						0.034
Never	38.1	47.0	48.4	48.6	41.2	
Ex-	38.7	39.6	38.3	39.5	44.0	
Current	23.2	13.4	13.4	11.9	14.8	
Age at diabetes diagnosis (years)	56.6 ± 12.6	57.0 ± 12.5	57.2 ± 10.8	58.2 ± 11.3	59.6 ± 11.6*	0.011
Diabetes duration (years)	3.2 [0.7–8.0]	4.0 [1.0–8.0]	4.0 [1.0–8.0]	4.0 [1.0–10.0]	4.0 [1.1–11.0]	0.19
Diabetes treatment (%)						0.07
Diet	27.3	28.0	33.9	31.3	35.1	
Oral glucose-lowering medications (OGLMs)	59.0	64.7	54.3	54.5	51.4	
Insulin ± OGLMs	13.7	7.3	11.8	14.2	13.5	
Fasting serum glucose (mmol/L)	8.6 [6.8–11.4]	8.7 [7.0–11.1]	8.3 [6.9–10.8]	8.5 [6.7–11.0]	8.2 [6.6–10.4]	0.43
HbA_1c_ (%)	7.7 [6.6–8.9]	7.4 [6.3–8.9]	7.4 [6.3–8.8]	7.6 [6.3–8.9]	7.3 [6.5–8.8]	0.90
HbA_1c_ (mmol/mol)	61 [49–74]	57 [45–74]	57 [45–73]	60 [45–74]	56 [48–73]	0.90
BMI (kg/m^2^)	30.2 ± 5.7	30.5 ± 5.8	29.8 ± 5.4	29.6 ± 5.2	28.5 ± 5.1**^,†††, ‡^	<0.001
Systolic blood pressure (mm Hg)	150 ± 26	149 ± 25	151 ± 24	149 ± 24	152 ± 22	0.32
Diastolic blood pressure (mm Hg)	81 ± 12	79 ± 12	81 ± 10	80 ± 11	80 ± 11	0.19
Antihypertensive medications (%)	48.1	46.6	51.9	42.7	59.1^†,###^	0.002
Diuretic therapy (%)	14.8	16.0	16.7	21.9	31.2***^,†††,‡‡‡^	<0.001
Serum potassium (mmol/L)	4.5 ± 0.4	4.5 ± 0.4	4.5 ± 0.4	4.5 ± 0.4	4.4 ± 0.5	0.19
Serum chloride (mmol/L)	105 ± 3	104 ± 3***	103 ± 3***^,††^	102 ± 2***^,†††,‡^	101 ± 3***^,†††,‡‡‡,##^	<0.001
Total serum cholesterol (mmol/L)	5.6 ± 1.5	5.5 ± 1.1	5.5 ± 1.0	5.5 ± 1.1	5.3 ± 1.0*	0.044
Serum HDL-cholesterol (mmol/L)	1.04 ± 0.30	1.02 ± 0.31	1.08 ± 0.33	1.06 ± 0.33	1.07 ± 0.33	0.21
Serum triglycerides (mmol/L)	2.2 (1.2–4.1)	2.1 (1.2–3.5)	1.9 (1.1–3.2)	1.8 (1.0–3.1)	1.7 (1.1–2.8)***^,††^	<0.001
Lipid-modifying treatment (%)	15.3	9.6	11.7	7.9	8.6	0.11
Aspirin therapy (% ≥75 mg/day)	18.6	21.9	20.9	19.7	25.5	0.35
Urinary albumin: creatinine ratio (mg/mmol)	3.3 (0.6–17.7)	3.3 (0.8–13.9)	2.9 (0.7–11.4)	3.0 (0.7–14.0)	3.1 (0.7–13.5)	0.86
eGFR (CKD-EPI) category (%)			*	*	***	<0.001
≥90 mL/min/1.73 m^2^	24.6	26.0	26.2	21.9	16.0	
60–89 mL/min/1.73 m^2^	44.8	51.1	54.1	55.6	56.9	
45–59 mL/min/1.73 m^2^	14.2	16.9	13.8	17.4	19.9	
30–44 mL/min/1.73 m^2^	9.8	4.6	4.4	4.5	6.4	
<30 mL/min/1.73 m^2^	6.6	1.4	1.5	0.6	0.8	
Peripheral sensory neuropathy (%)	26.7	34.6	27.6	33.3	32.9	0.24
Peripheral arterial disease (%)	28.5	26.2	27.1	25.1	35.7	0.041
Coronary heart disease (%)	26.2	29.7	27.3	22.5	36.5^#^	0.007
Cerebrovascular disease (%)	9.3	8.2	9.7	6.2	13.8	0.06
Charlson comorbidity index (%)						0.06
0	71.6	71.7	74.2	79.2	64.9	
1–2	21.3	21.9	20.2	17.4	26.8	
≥3	7.1	6.4	5.6	3.4	8.3	

* P < 0.05

** P < 0.01

*** P < 0.001 vs Q1

^†^ *P* < 0.05

^††^ *P* < 0.01

^†††^ *P* < 0.001 vs Q2

^‡^ *P* < 0.05

^‡‡^ *P* < 0.01

^‡‡‡^ *P* < 0.001 vs Q3

^#^ *P* < 0.05

^##^ *P* < 0.01

^###^ *P* < 0.001 vs Q3—pairwise comparisons adjusted for multiple comparisons using the Bonferroni correction

**Table 2:** Multiple linear regression model of independent associates of baseline serum bicarbonate in patients with type 2 diabetes

	Coefficient	*P* value
Male	0.97	<0.001
Ex-smoker	−0.35	0.038
Age at diagnosis (increase of 1 year)	0.02	0.007
Insulin therapy	0.87	0.001
Diuretic therapy	0.50	0.023
Total serum cholesterol (per 1 mmol/L increase)	0.22	0.006
Log_e_(serum triglycerides) (per increase of 1)^a^	−0.59	<0.001
Serum chloride (per 1 mmol/L increase)	−0.20	<0.001
Coronary heart disease	−0.39	0.045
Charlson comorbidity index^b^		
0 (reference)	0	
1 or 2	−0.42	0.049
≥3	−1.06	0.006

^a^A 2.72-fold increase in serum triglycerides corresponds to an increase of 1 in log_e_(serum triglycerides)

^b^In the previous 5 years, excluding diabetes and its complications

There were 383 (29.6 %) participants with type 2 diabetes who had a prior history of CHD at study entry and 111 (8.6 %) who had been hospitalized previously for/with HF. These patients were excluded from the respective analyses of incident CHD and HF.

### Serum bicarbonate and mortality

During a total of 16,769 patient-years (12.9 ± 6.1 years) of follow-up to end-December 2012, 738 (56.9 %) of the cohort died. In unadjusted analysis, patients in the lowest quintile of serum bicarbonate were at a significantly increased risk of death compared to those in the highest quintile (see Table 3). In the fully adjusted model, patients in the lowest quintile of serum bicarbonate were not at increased risk. There was no significant association between serum bicarbonate analyzed as a continuous variable and mortality in either unadjusted or fully adjusted analyses (Table 3).

**Table 3:** Hazard ratio (95 % CI) of serum bicarbonate concentration as quintiles and as a continuous variable for all-cause mortality, incident coronary heart disease and incident heart failure in patients with type 2 diabetes unadjusted or adjusted for the respective most parsimonious Cox proportional hazards models

	Serum bicarbonate quintile (mmol/L)					
	Q1 ≤23	Q2 24–25	Q3 26–27	Q4 28	Q5 ≥29	All increase of 1
All-cause mortality						
Number	183	219	341	178	362	1283^a^
Number of events (%)	104 (56.8)	122 (55.7)	182 (53.4)	97 (54.5)	227 (62.7)	732 (57.1)
Unadjusted	1.27 (1.004–1.60)	1.07 (0.86–1.33)	1.02 (0.84–1.24)	1.01 (0.80–1.29)	1.00 (reference)	0.98 (0.96–1.004)
Adjusted^b^	1.25 (0.96–1.63)	1.07 (0.83–1.38)	1.04 (0.83–1.29)	1.10 (0.84–1.43)	1.00 (reference)	0.98 (0.95–1.004)
Coronary heart disease						
Number	135	154	248	138	230	905^c^
Number of events (%)	59 (43.7)	70 (45.5)	93 (37.5)	50 (36.2)	89 (38.7)	361 (39.9)
Unadjusted	1.58 (1.14–2.20)	1.53 (1.12–2.10)	1.14 (0.86–1.53)	0.97 (0.68–1.37)	1.00 (reference)	0.94 (0.91–0.97)
Adjusted^d^	1.39 (0.97–1.98)	1.23 (0.88–1.72)	0.97 (0.71–1.31)	0.92 (0.64–1.32)	1.00 (reference)	0.95 (0.92–0.99)
Heart failure						
Number	172	200	325	167	310	1174^e^
Number of events (%)	66 (38.4)	58 (29.0)	98 (30.2)	44 (26.3)	108 (34.8)	374 (31.9)
Unadjusted	1.54 (1.13–2.09)	1.04 (0.76–1.43)	1.05 (0.80–1.38)	0.81 (0.57–1.15)	1.00 (reference)	0.95 (0.92–0.98)
Adjusted^f^	1.08 (0.76–1.53)	0.82 (0.58–1.16)	0.90 (0.67–1.21)	0.77 (0.52–1.13)	1.00 (reference)	0.99 (0.95–1.03)

^a^13 participants had missing serum bicarbonate

^b^Adjusted for age as time scale, age at baseline, sex, Aboriginal, current smoking status, any exercise in the past 2 weeks, ACE-inhibitor use, diuretic use, diabetes duration, on lipid-modifying medication, log_e_(urinary albumin:creatinine ratio), retinopathy, peripheral sensory neuropathy, peripheral arterial disease, coronary heart disease, comorbidities (Charlson Comorbidity Index excluding diabetes and its complications)

^c^383 had a history of CHD at baseline, 8 had missing serum bicarbonate

^d^Adjusted for age as time scale, age at baseline, sex, not fluent in English, diabetes duration, log_e_(serum triglycerides), log_e_(urinary albumin:creatinine ratio), peripheral sensory neuropathy, peripheral arterial disease

^e^111 had a history of HF at baseline, 11 had missing serum bicarbonate

^f^Adjusted for age as time scale, age at baseline, currently married/de facto relationship, any exercise in the past 2 weeks, on digoxin, log_e_(urinary albumin:creatinine ratio), eGFR (CKD-EPI) ≥90 or <30 mL/min/1.73 m^2^, retinopathy, peripheral sensory neuropathy, coronary heart disease, cerebrovascular disease, schizophrenia

### Serum bicarbonate and coronary heart disease

**Fig. 1:**
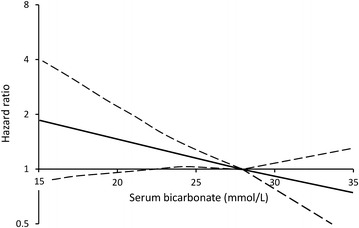
Relationship between serum bicarbonate and risk of incident coronary heart disease in patients with type 2 diabetes without CHD at baseline shown by restricted cubic spline modeling (*solid line* with reference 28 mmol/L and the optimum 3 knots) and 95 % confidence intervals (*dashed lines*) after adjustment for age as time scale, age at baseline, sex, fluency in English, diabetes duration, log_e_(serum triglycerides), log_e_(urinary albumin: creatinine ratio), peripheral sensory neuropathy and peripheral arterial disease

During 10,540 patient-years (11.5 ± 6.3 years) of follow-up to end-June 2012, 365 (39.9 %) of the cohort with no CHD at study entry had a CHD event. In unadjusted analysis, patients with serum bicarbonate ≤25 mmol/L were at increased risk of CHD compared to those in the highest quintile (see Table 3). After adjustment for the most parsimonious Cox regression model, this result was no longer significant. There was an association between serum bicarbonate as a continuous variable and CHD in unadjusted analysis, and after adjustment this remained significant. The risk of an incident CHD event decreased by 5 % for each 1 mmol/L increase in serum bicarbonate. Restricted cubic spline modeling confirmed that the increase in risk as bicarbonate fell was linear and negative, with no indication of a change in the slope of the relationship at higher bicarbonate concentrations (see Fig. 1).

### Serum bicarbonate and heart failure

During 14,393 patient-years (12.1 ± 6.2 years) of follow-up to end-June 2012, 377 (31.8 %) participants with T2DM with no HF at study entry, suffered incident HF. Patients in the lowest quintile of serum bicarbonate were at increased risk of a congestive heart failure event in unadjusted analysis, but again, after adjustment for relevant covariates, this association was non-significant (see Table 3). When analysed as a continuous variable, serum bicarbonate was associated with incident HF events in unadjusted analysis, but this relationship was no longer significant after adjustment.

### Alternative multivariable analyses

Associations between serum bicarbonate and mortality, CHD and HF were re-examined using a pre-defined set of co-variates applied in a step-wise manner rather than the most-parsimonious models described above (see Additional file 3). There was no change in the outcomes.

## Discussion

In our community-based representative patients with type 2 diabetes, there was a significant independent inverse association between baseline serum bicarbonate and the risk of incident CHD in patients without a history CHD at baseline. Patients in the lowest quintiles of serum bicarbonate were at increased risk of death and incident HF, but adjustment for clinically relevant covariates in the most parsimonious models rendered both these associations non-significant. These results indicate that serum bicarbonate is a significant predictor of incident CHD in type 2 diabetes, but that it acts as a surrogate for other pathological processes underlying HF and all-cause mortality.

We observed an increased risk of all-cause mortality in those with serum bicarbonate ≤23 mmol/L compared to those in the highest quintile in unadjusted analysis during a mean of 12.9 years of follow-up, but this did not remain significant after adjustment for a large range of other explanatory variables. The only other study of the association between serum bicarbonate and mortality exclusively in diabetes involved combined data from patients with CKD who participated in the Reduction of End points in Non-insulin-dependent diabetes with the Angiotensin II Antagonist Losartan (RENAAL) trial or the Irbesartan Diabetic Nephropathy Trial (IDNT) [10]. There was increased mortality during a median 2.8 years of follow-up in patients with serum bicarbonate in the lowest two quartiles that was no longer significant after full adjustment for other variables including eGFR. Similarly, significant bivariate associations between baseline bicarbonate and mortality in two other studies of CKD patients from the general population were also no longer present after adjustment [2, 22]. Our observations are consistent with these studies in showing no independent association between serum bicarbonate and death.

Other studies in samples of generally healthy subjects and in CKD patients have, by contrast, demonstrated significant and independent associations between serum bicarbonate and all-cause mortality [3–8]. In the Health ABC study of people aged 70–79 years [7], a baseline arterialized venous blood serum bicarbonate <23.0 mmol/L was independently associated with increased mortality compared to 23.0–27.9 mmol/L. National Health and Nutrition Examination Survey III data from a cohort of 15,836 participants selected to represent the general US population also showed that a low serum bicarbonate (<22 mmol/L) was associated with mortality independently of age, gender, race, eGFR, albuminuria, cardiovascular disease, lung disease, diabetes, hypertension, smoking status, C-reactive protein and estimated protein intake normalized to body weight [8]. In a Korean study of the health screening records from 31,590 participants, there was an independent increased mortality risk for those with a serum bicarbonate in the lowest compared to the highest quartile [6]. In two clinic-based studies of non-dialysis CKD patients, there was also an independent increased mortality risk for those with low serum bicarbonate concentrations [4, 5] in addition to increased mortality in the highest serum bicarbonate category (>32 mmol/L) in one [5]. In a second analysis of the Chronic Renal Insufficiency Cohort (CRIC) study involving CKD patients not on hemodialysis, there was an independent association between a high updated mean serum bicarbonate and mortality, but no independent association for a low serum bicarbonate [3].

By contrast, three studies of community-based participants and two carried out in clinic-based samples of non-dialysis dependent CKD patients found independent associations between serum bicarbonate and mortality. The apparent discrepancy between studies based on the diabetes status of the participants may relate to the availability of covariates. Factors such as exercise and comorbidity were significantly associated with mortality in the most parsimonious regression model in our study but were typically not available for use in multivariable mortality models in general population studies. Alternatively, it may be that the pathophysiological changes associated with diabetes swamp a potential contribution from serum bicarbonate. This would be consistent with the observation in several studies that, while serum bicarbonate was associated with death in the sample as a whole, there was no such association in the sub-groups of patients with diabetes [4, 5]. This finding, the RENAAL/IDNT study data [10] and our own observations all suggest that a low serum bicarbonate is not an independent risk factor for all-cause mortality in type 2 diabetes.

In contrast to mortality, we found a significant independent inverse association between serum bicarbonate and first incident CHD event, with a linear 5 % reduction in risk per 1 mmol/L increase in serum bicarbonate. For an FDS patient with serum bicarbonate of 22 mmol/L, the lower limit of the laboratory reference interval, the relative risk of CHD was approximately 25 % greater that of a patient with a serum bicarbonate at the mean concentration in the total sample of 27 mmol/L. Three reports from two other studies have also assessed this association, specifically the combined RENAAL/IDNT study of patients with diabetic nephropathy [10] and the CRIC study [2, 3], but there was no independent association between serum bicarbonate and atherosclerotic events in either study after median durations of follow-up of 2.8, 3.9 and 6 years, respectively. The cardiovascular events in these studies included not only myocardial infarction but also stroke and, in the CRIC cohort, peripheral vascular disease. This heterogeneous endpoint may have masked a specific association between serum bicarbonate and CHD. Furthermore, prior cardiovascular disease was not included as a covariate in the RENAAL/IDNT study [10] and in the earlier CRIC analysis [2]. The longer follow-up in the present study (with a resultant higher proportion of patients experiencing CHD than in the RENAAL/IDNT and CRIC studies), the more specific end-point, and our exclusion of patients with prevalent CHD may explain the greater sensitivity of the present study to detect an association between serum bicarbonate and incident CHD risk.

We observed a significant increase in risk of HF in our patients with a serum bicarbonate in the lowest quintile, and a significant inverse association between incident HF and serum bicarbonate when analysed as a continuous variable. However these associations were lost in the fully adjusted model. Of note, however, the lowest risk appeared to be in the 4th rather than the 5th quintile, suggesting the possibility of a U-shaped relationship, although this difference was not significant. In contrast to our observations, two studies have found significant positive associations between measures of serum bicarbonate and incident HF [3, 9]. One study found a significant positive association between updated mean bicarbonate and incident HF in patients with CKD, and those with mean bicarbonate >26 mmol/L had an independent 66 % increased risk compared to those with mean bicarbonate of 22–26 mmol/L [3]. Similarly, a second study found that those in the highest quartile of bicarbonate had an increased risk of HF hospitalization than those in the 3rd quartile after adjustment, but not in the unadjusted model [10]. In neither study was a low serum bicarbonate associated with incident HF. It may be that there is a difference between the patients in these studies that modulates the association between bicarbonate and HF. For example, the patients in the previous studies [3, 10] had relatively poor renal function compared to those in the present study and the adjustments in previous studies included many variables associated with serum bicarbonate, but they did not include recent exercise and digoxin use which may reflect increased HF risk and which we found to be significant covariates in the most parsimonious model. As noted above, the analysis from the RENAAL/IDNT study did not include adjustments for prevalent cardiovascular and cerebrovascular disease [10] while the use of updated mean serum bicarbonate may not be directly comparable to baseline serum bicarbonate in an ageing patient [3]. We conclude that serum bicarbonate is not a significant risk factor for HF in community-dwelling patients with type 2 diabetes.

We examined the independent cross-sectional associates of serum bicarbonate to shed light on factors which may be influential and which may need to be considered as covariates in the prospective analyses. Serum chloride was negatively associated, consistent with known mechanisms that maintain electrochemical neutrality and systemic pH. Diuretic therapy was positively associated with serum bicarbonate, probably explained by mild potassium loss and a tendency towards metabolic alkalosis in these patients. The negative association between serum bicarbonate with serum triglycerides may be due to associated higher plasma fatty acid or ketone body concentrations in those with higher serum triglycerides, tending to cause a mild metabolic acidosis. Interestingly, although eGFR category was significantly associated with quintile of serum bicarbonate in univariate analysis (see Table 1), it was not associated in the multivariate model. This may reflect a U-shaped relationship between lower bicarbonate category and eGFR, with higher proportions of patients in lower bicarbonate quintiles in both the highest and lowest eGFR categories.

The question arises as to why a low serum bicarbonate appears to be an independent risk factor for CHD in type 2 diabetes. The independent associations between both serum triglycerides and insulin therapy and serum bicarbonate in our cohort suggest a possible relationship between bicarbonate and insulin resistance. Supportive evidence for this possibility comes from two sources. Insulin sensitivity in clamp studies was independently associated with serum bicarbonate and apolipoprotein A1: apolipoprotein B ratio in a sample of CKD patients without diabetes [23]. Experimental metabolic acidosis in healthy humans in vivo and acidification of the medium in which adipocytes were cultured in vitro leads to reductions in serum adiponectin and adiponectin mRNA production, respectively [24]. Therefore, metabolic acidosis may worsen insulin resistance and thereby increase CHD risk. An alternative explanation may be that the low serum bicarbonate associated with CHD reflects a compensated respiratory alkalosis due to impaired lung function. Type 2 diabetes is associated with a 10–20 % reduction in measures of lung function that was associated with prevalent CHD in a sub-group of FDS patients [25] while mild respiratory alkalosis has been associated with mortality in a sample of healthy older people [7]. Nevertheless, in the absence of a measure of systemic pH we are unable to say whether relative acidosis or alkalosis is associated with CHD in our cohort. Further studies are required to determine how serum bicarbonate is associated with CHD in diabetes.

A recent systematic review of the few trials of oral bicarbonate therapy in patients with CKD confirmed preservation of renal function and reduced incidence of progression to renal replacement therapy in bicarbonate-treated patients, and recommended that large, well-controlled trials of bicarbonate treatment should be carried out [26]. Although some guidelines for CKD management include oral alkalinization therapy for selected patients, current US and Australian guidelines for prevention and management of CKD in type 2 diabetes do not recommend bicarbonate therapy [1, 27]. Our observation of an independent association between serum bicarbonate and CHD risk adds impetus to calls for high quality trials of bicarbonate therapy with mortality and cardiovascular end-points, as well as renal outcomes [28, 29]. Our data suggest that enrolling those with bicarbonate concentrations ≤25 mmol/L would include the majority of patients at increased risk. Until this evidence becomes available, patients with diabetes should be encouraged to follow diets rich in fruit and vegetables as a means of providing alkalinization therapy that is safe and as effective as oral sodium bicarbonate in preserving renal function [30]. For patients with diabetes who have a serum bicarbonate <22 mmol/L, measurement of venous pH to assess acidosis status appears prudent before more definitive alkalinization therapy is suggested.

### Limitations of the present study

Serum bicarbonate concentrations were reported in whole numbers (as is usual laboratory practice) which limited the ability to define exact quintile boundaries. Our analyses were based on a single serum bicarbonate measurement and so the contribution of changes in serum bicarbonate to outcome were not captured. We did not have simultaneous pH measurements to allow us to establish acid–base status. Although we had a wide range of plausible confounding factors available to use as covariates in the multivariable analysis it is possible that residual confounding from some unmeasured factor remained. Our cohort did not have sufficient cases of incident end-stage renal disease to allow a meaningful assessment of this outcome. The strengths of the present study include the long duration of follow-up, the range of covariates available for analysis, and the near complete ascertainment of mortality and other endpoints from active ascertainment and linkage to the WADLS.

## Conclusions

We found a significant inverse and independent association between serum bicarbonate with incident CHD in community-based patients with type 2 diabetes without a previous history of CHD. This observation supports future adequately powered clinical trials of bicarbonate replacement in high risk patients with type 2 diabetes and a low serum bicarbonate, including those with CKD.

## Additional files

10.1186/s12933-016-0462-x Baseline characteristics by survival of FDS patients to the end of 2012. **Table S2.** Baseline characteristics by incident coronary heart disease (CHD) status by end-June 2012 in FDS patients with type 2 diabetes and no history of CHD. **Table S3.** Baseline characteristics by incident heart failure (HF) status by end-June 2012 in FDS patients with type 2 diabetes and no history of HF.

10.1186/s12933-016-0462-x Most parsimonious Cox proportional hazards models (excluding serum bicarbonate) of all-cause mortality, incident coronary heart disease (CHD) and incident heart failure (HF) with age as time scale in FDS patients.

10.1186/s12933-016-0462-x Hazard ratios and cause-specific hazard ratios (95% CI) of serum bicarbonate concentration as quintiles and as a continuous variable for all-cause mortality, incident coronary heart disease and incident heart failure in patients with type 2 diabetes with age as time scale. (A) unadjusted; (B) adjusted for age and sex; (C) adjusted for (B) plus Aboriginal, current smoking status, any exercise in the past two weeks, not fluent in English, currently married/de facto relationship, BMI; (D) adjusted for (C) plus systolic and diastolic blood pressures, HbA1c, total and HDL-cholesterol, ln(serum triglycerides); and (E) adjusted for (D) plus ln(urinary albumin:creatinine ratio), eGFR (CKD-EPI) ≥90 or <30 ml/min/1.73m2, retinopathy, peripheral sensory neuropathy, coronary heart disease and cerebrovascular disease.

## Abbreviations

CCI: Charlson comorbidity index
CKD: chronic kidney disease
CKD-EPI: chronic kidney disease epidemiology collaboration
CHD: coronary heart disease
eGFR: estimated glomerular filtration rate
FDS: Fremantle diabetes study
HF: heart failure
IDNT: irbesartan diabetic nephropathy trial
RENAAL: reduction of end points in non-insulin-dependent diabetes with the angiotensin ii antagonist losartan trial
WA: Western Australia
WADLS: Western Australian data linkage system

## References

[1] American Diabetes Association (2016). Microvascular complications and foot care. Diabetes Care.

[2] Dobre M, Yang W, Chen J, Drawz P, Hamm LL, Horwitz E, Hostetter T, Jaar B, Lora CM, Nessel L (2013). Association of serum bicarbonate with risk of renal and cardiovascular outcomes in CKD: a report from the Chronic Renal Insufficiency Cohort (CRIC) study. Am J Kidney Dis.

[3] Dobre M, Yang W, Pan Q, Appel L, Bellovich K, Chen J, Feldman H, Fischer MJ, Ham LL, Hostetter T (2015). Persistent high serum bicarbonate and the risk of heart failure in patients with chronic kidney disease (CKD): a report from the chronic renal insufficiency cohort (CRIC) study. J Am Heart Assoc.

[4] Kovesdy CP, Anderson JE, Kalantar-Zadeh K (2009). Association of serum bicarbonate levels with mortality in patients with non-dialysis-dependent CKD. Nephrol Dial Transplant.

[5] Navaneethan SD, Schold JD, Arrigain S, Jolly SE, Wehbe E, Raina R, Simon JF, Srinivas TR, Jain A, Schreiber MJ (2011). Serum bicarbonate and mortality in stage 3 and stage 4 chronic kidney disease. Clin J Am Soc Nephrol.

[6] Park M, Jung SJ, Yoon S, Yun JM, Yoon HJ (2015). Association between the markers of metabolic acid load and higher all-cause and cardiovascular mortality in a general population with preserved renal function. Hypertens Res.

[7] Raphael KL, Murphy RA, Shlipak MG, Satterfield S, Huston HK, Sebastian A, Sellmeyer DE, Patel KV, Newman AB, Sarnak MJ (2016). Bicarbonate concentration, acid-base status, and mortality in the health, aging, and body composition study. Clin J Am Soc Nephrol.

[8] Raphael KL, Zhang Y, Wei G, Greene T, Cheung AK, Beddhu S (2013). Serum bicarbonate and mortality in adults in NHANES III. Nephrol Dial Transplant.

[9] Horne BD, May HT, Muhlestein JB, Ronnow BS, Lappe DL, Renlund DG, Kfoury AG, Carlquist JF, Fisher PW, Pearson RR (2009). Exceptional mortality prediction by risk scores from common laboratory tests. Am J Med.

[10] Schutte E, Lambers Heerspink HJ, Lutgers HL, Bakker SJ, Vart P, Wolffenbuttel BH, Umanath K, Lewis JB, de Zeeuw D, Gansevoort RT (2015). Serum bicarbonate and kidney disease progression and cardiovascular outcome in patients with diabetic nephropathy: a post hoc analysis of the RENAAL (reduction of end points in non-insulin-dependent diabetes with the angiotensin ii antagonist losartan) Study and IDNT (Irbesartan diabetic nephropathy trial). Am J Kidney Dis.

[11] Driver TH, Shlipak MG, Katz R, Goldenstein L, Sarnak MJ, Hoofnagle AN, Siscovick DS, Kestenbaum B, de Boer IH, Ix JH (2014). Low serum bicarbonate and kidney function decline: the multi-ethnic study of atherosclerosis (MESA). Am J Kidney Dis.

[12] Shah SN, Abramowitz M, Hostetter TH, Melamed ML (2009). Serum bicarbonate levels and the progression of kidney disease: a cohort study. Am J Kidney Dis.

[13] Davis TM, Bruce DG, Davis WA (2013). Cohort profile: the Fremantle Diabetes Study. Int J Epidemiol.

[14] Australian Bureau of Statistics. Socio-economic indexes for areas. 2013. http://www.abs.gov.au/websitedbs/censushome.nsf/home/seifa. Accessed Aug 2016.

[15] Norman PE, Davis WA, Bruce DG, Davis TM (2006). Peripheral arterial disease and risk of cardiac death in type 2 diabetes: the Fremantle Diabetes Study. Diabetes Care.

[16] Feldman EL, Stevens MJ, Thomas PK, Brown MB, Canal N, Greene DA (1994). A practical two-step quantitative clinical and electrophysiological assessment for the diagnosis and staging of diabetic neuropathy. Diabetes Care.

[17] Levey AS, Stevens LA, Schmid CH, Zhang YL, Castro AF, Feldman HI, Kusek JW, Eggers P, Van Lente F, Greene T (2009). A new equation to estimate glomerular filtration rate. Ann Intern Med.

[18] Charlson ME, Pompei P, Ales KL, MacKenzie CR (1987). A new method of classifying prognostic comorbidity in longitudinal studies: development and validation. J Chronic Dis.

[19] Holman CD, Bass AJ, Rosman DL, Smith MB, Semmens JB, Glasson EJ, Brook EL, Trutwein B, Rouse IL, Watson CR (2008). A decade of data linkage in Western Australia: strategic design, applications and benefits of the WA data linkage system. Aust Health Rev.

[20] Eckerbom S, Bergqvist Y, Jeppsson JO (1994). Improved method for analysis of glycated haemoglobin by ion exchange chromatography. Ann Clin Biochem.

[21] Thiebaut AC, Benichou J (2004). Choice of time-scale in Cox’s model analysis of epidemiologic cohort data: a simulation study. Stat Med.

[22] Menon V, Tighiouart H, Vaughn NS, Beck GJ, Kusek JW, Collins AJ, Greene T, Sarnak MJ (2010). Serum bicarbonate and long-term outcomes in CKD. Am J Kidney Dis.

[23] Kobayashi S, Maesato K, Moriya H, Ohtake T, Ikeda T (2005). Insulin resistance in patients with chronic kidney disease. Am J Kidney Dis.

[24] Disthabanchong S, Niticharoenpong K, Radinahamed P, Stitchantrakul W, Ongphiphadhanakul B, Hongeng S (2011). Metabolic acidosis lowers circulating adiponectin through inhibition of adiponectin gene transcription. Nephrol Dial Transplant.

[25] Davis TM, Knuiman M, Kendall P, Vu H, Davis WA (2000). Reduced pulmonary function and its associations in type 2 diabetes: the Fremantle Diabetes Study. Diabetes Res Clin Pract.

[26] Susantitaphong P, Sewaralthahab K, Balk EM, Jaber BL, Madias NE (2012). Short-and long-term effects of alkali therapy in chronic kidney disease: a systematic review. Am J Nephrol.

[27] Chadban S, Howell M, Twigg S, Thomas M, Jerums G, Cass A, Campbell D, Nicholls K, Tong A, Mangos G (2010). The CARI guidelines. Prevention and management of chronic kidney disease in type 2 diabetes. Nephrology.

[28] Di Iorio B, Aucella F, Conte G, Cupisti A, Santoro D (2012). A prospective, multicenter, randomized, controlled study: the correction of metabolic acidosis with use of bicarbonate in Chronic Renal Insufficiency (UBI) Study. J Nephrol.

[29] Gaggl M, Cejka D, Plischke M, Heinze G, Fraunschiel M, Schmidt A, Horl WH, Sunder-Plassmann G (2013). Effect of oral sodium bicarbonate supplementation on progression of chronic kidney disease in patients with chronic metabolic acidosis: study protocol for a randomized controlled trial (SoBic-Study). Trials.

[30] Goraya N, Simoni J, Jo CH, Wesson DE (2013). A comparison of treating metabolic acidosis in CKD stage 4 hypertensive kidney disease with fruits and vegetables or sodium bicarbonate. Clin J Am Soc Nephrol.

