# Supplementary material for: Serum bicarbonate concentration and the risk of cardiovascular disease and death in type 2 diabetes: the Fremantle Diabetes Study
Source: Cardiovasc Diabetol. 2016 Oct 6;15:143. doi: 10.1186/s12933-016-0462-x (PMC5054557; doi:10.1186/s12933-016-0462-x)
Supplement: Supplementary file 1 — 10.1186/s12933-016-0462-x Baseline characteristics by survival of FDS patients to the end of 2012. Table S2. Baseline characteristics by incident coronary heart disease (CHD) status by end-June 2012 in FDS patients with type 2 diabetes and no history of CHD. Table S3. Baseline characteristics by incident heart failure (HF) status by end-June 2012 in FDS patients with type 2 diabetes and no history of HF. [file 12933_2016_462_MOESM1_ESM.docx]

**Additional Table 1.** Baseline characteristics by survival of FDS patients to the end of 2012.

|  | Deceased | Alive | *P*-value |
| --- | --- | --- | --- |
| Number (%) | 738 (56.9) | 558 (43.1) |  |
| Age at study entry (years) | 69.3±8.9 | 56.9±10.1 | <0.001 |
| Sex (% male) | 52.8 | 43.0 | <0.001 |
| Ethnic background (%): |  |  | <0.001 |
| Anglo-Celt | 65.2 | 56.5 |  |
| Southern European | 17.1 | 18.6 |  |
| Other European | 8.9 | 7.9 |  |
| Asian | 1.8 | 5.6 |  |
| Indigenous Australian | 1.6 | 1.3 |  |
| Mixed/other | 5.4 | 10.2 |  |
| Not fluent in English (%) | 16.0 | 14.3 | 0.44 |
| Education beyond primary level (%) | 70.9 | 78.1 | 0.004 |
| Currently married/de facto (%) | 60.5 | 72.4 | <0.001 |
| Alcohol (standard drinks/day) | 0 [0-0.8] | 0 [0-0.7] | 0.58 |
| Smoking status (% never/ex/current) | 40.8/42.8/16.4 | 49.8/36.8/13.4 | 0.005 |
| Any exercise in past 2 weeks (%) | 67.4 | 77.8 | <0.001 |
| Age at diagnosis (years) | 69.4±8.9 | 56.9±10.1 | <0.001 |
| Duration of diabetes (years) | 5.0 [1.9-11.0] | 2.4 [0.5-6.0] | <0.001 |
| Diabetes treatment (%): |  |  | <0.001 |
| Diet | 27.0 | 38.5 |  |
| Oral agents | 57.7 | 53.7 |  |
| Insulin ± oral agents | 15.3 | 7.8 |  |
| Fasting glucose (mmol/L) | 8.5 [6.9-10.80] | 8.3 [6.8-10.7] | 0.29 |
| HbA1c_1c_ (%) | 7.6 [6.6-9.0] | 7.2 [6.2-8.6] | <0.001 |
| HbA_1c_ (mmol/mol) | 60 [49-75] | 55 [44-70] | <0.001 |
| BMI (kg/m^2^) | 29.0±5.1 | 30.3±5.7 | <0.001 |
| Obesity (% by waist circumference^¶^) | 63.9 | 65.5 | 0.56 |
| Systoli blood pressure (mm Hg) | 157±264 | 143±21 | <0.001 |
| Diastolic blood pressure (mm Hg) | 81±12 | 80±10 | 0.023 |
| Antihypertensive medication (%) | 59.8 | 39.2 | <0.001 |
| Angiotensin converting enzyme inhibitor | 15.4 | 26.8 | <0.001 |
| Beta-blocker | 13.1 | 17.4 | 0.036 |
| Calcium channel blocker | 13.4 | 24.2 | <0.001 |
| Diuretic | 11.8 | 27.9 | <0.001 |
| Serum potassium (mmol/L) | 4.4±0.4 | 4.5±0.5 | <0.001 |
| Serum chloride (mmol/L) | 103±3 | 103±3 | 0.030 |
| Serum bicarbonate (mmol/L) | 27±3 | 27±3 | 0.15 |
| Total serum cholesterol (mmol/L) | 5.4±1.1 | 5.5±1.1 | 0.10 |
| Serum HDL-cholesterol (mmol/L) | 1.07±0.34 | 1.05±0.30 | 0.23 |
| Serum triglycerides (mmol/L) | 1.9 (1.1-3.3) | 1.9 (1.1-3.3) | 0.34 |
| Lipid-modifying medication (%) | 9.4 | 12.0 | 0.14 |
| Aspirin use (%) | 26.7 | 15.3 | <0.001 |
| Urinary albumin:creatinine (mg/mmol) | 4.8 (1.0-22.5) | 1.7 (0.6-5.4) | <0.001 |
| eGFR categories (%): |  |  | <0.001 |
| ≥90 ml/min/1.73m^2^ | 13.4 | 34.4 |  |
| 60-89 ml/min/1.73m^2^ | 52.1 | 54.9 |  |
| 45-59 ml/min/1.73m^2^ | 21.8 | 9.8 |  |
| 30-44 ml/min/1.73m^2^ | 9.6 | 0.7 |  |
| <30 ml/min/1.73m^2^ | 3.1 | 0.2 |  |
| Coronary heart disease (%) | 39.2 | 16.8 | <0.001 |
| Cerebrovascular disease (%) | 14.6 | 3.8 | <0.001 |
| Peripheral arterial disease (%) | 40.1 | 15.5 | <0.001 |
| Peripheral sensory neuropathy (%) | 41.7 | 16.9 | <0.001 |
| Retinopathy (%) | 21.1 | 10.4 | <0.001 |
| Charlson Comorbidity Index: |  |  | <0.001 |
| 0 | 60.4 | 86.2 |  |
| 1-2 | 29.7 | 12.0 |  |
| 3+ | 9.9 | 1.8 |  |
| Schizophrenia (%) | 0.7 | 0 | 0.07 |

^¶^Waist circumference ≥102 cm in men and ≥88 cm in women

**Additional Table 2.** Baseline characteristics by incident coronary heart disease (CHD) status by end-June 2012 in FDS patients with type 2 diabetes and no history of CHD.

|  | No incident CHD | Incident CHD | *P*-value |
| --- | --- | --- | --- |
| Number (%) | 549 (60.1) | 364 (39.9) |  |
| Age (years) | 60.9±11.9 | 65.0±11.0 | <0.001 |
| Male (%) | 43.7 | 49.7 | 0.08 |
| Ethnic background (%): |  |  | 0.14 |
| Anglo-Celt | 58.8 | 64.8 |  |
| Southern European | 20.2 | 16.2 |  |
| Other European | 8.2 | 7.4 |  |
| Asian | 4.6 | 1.9 |  |
| Aboriginal | 1.1 | 1.4 |  |
| Mixed/other | 7.1 | 8.2 |  |
| Not fluent in English (%) | 19.3 | 12.4 | 0.006 |
| Educated beyond primary school (%) | 72.5 | 76.3 | 0.22 |
| Currently married/*de facto* relationship (%) | 68.7 | 62.4 | 0.05 |
| Alcohol consumption (standard drinks/day) | 0 [0-0.8] | 0 [0-0.3] | 0.51 |
| Smoking status (%): |  |  | 0.013 |
| Never | 51.5 | 41.5 |  |
| Ex- | 34.4 | 40.9 |  |
| Current | 14.1 | 17.5 |  |
| Age at diabetes diagnosis (years) | 56.0±12.0 | 58.4±11.4 | 0.003 |
| Diabetes duration (years) | 3.0 [0.6-7.0] | 4.0 [1.0-10.0] | <0.001 |
| Diabetes treatment (%): |  |  | 0.006 |
| Diet | 36.7 | 28.1 |  |
| Oral glucose-lowering medications (OGLMs) | 54.8 | 58.7 |  |
| Insulin±OGLMs | 8.4 | 13.2 |  |
| Fasting serum glucose (mmol/L) | 8.3 [6.7-10.5] | 8.7 [7.0-11.0] | 0.11 |
| HbA_1c_ (%) | 7.2 [6.2-8.5] | 7.7 [6.6-8.9] | 0.001 |
| HbA_1c_ (mmol/mol) | 55 [44-69] | 61 [49-74] | 0.001 |
| BMI (kg/m^2^) | 29.8±5.9 | 29.4±5.3 | 0.27 |
| Systolic blood pressure (mm Hg) | 147±23 | 154±24 | <0.001 |
| Diastolic blood pressure (mm Hg) | 80±11 | 82±11 | 0.05 |
| On antihypertensive medications (%) | 38.4 | 44.8 | 0.06 |
| On angiotensin converting enzyme inhibitors (%) | 16.6 | 21.5 | 0.07 |
| On diuretics (%) | 16.9 | 15.4 | 0.58 |
| Serum potassium (mmol/L) | 4.4±0.4 | 4.5±0.4 | 0.005 |
| Serum chloride (mmol/L) | 103±3 | 103±3 | 0.82 |
| Total serum cholesterol (mmol/L) | 5.4±1.0 | 5.6±1.2 | 0.026 |
| Serum HDL-cholesterol (mmol/L) | 1.09±0.33 | 1.06±0.31 | 0.11 |
| Serum triglycerides (mmol/L) | 1.8 (1.0-3.0) | 1.9 (1.1-3.4) | 0.021 |
| On lipid-modifying treatment (%) | 7.8 | 6.0 | 0.36 |
| On aspirin (% ≥75 mg/day) | 8.2 | 13.3 | 0.018 |
| Serum bicarbonate (mmol/L) | 27±3 | 27±3 | 0.18 |
| Urinary albumin:creatinine ratio (mg/mmol) | 2.5 (0.6-10.1) | 3.5 (0.8-15.2) | <0.001 |
| eGFR category (%): |  |  | 0.003 |
| ≥90 ml/min/1.73m^2^ | 30.3 | 22.9 |  |
| 60-89 ml/min/1.73m^2^ | 54.2 | 52.2 |  |
| 45-59 ml/min/1.73m^2^ | 10.3 | 18.0 |  |
| 30-44 ml/min/1.73m^2^ | 3.3 | 5.2 |  |
| <30 ml/min/1.73m^2^ | 1.8 | 1.7 |  |
| Peripheral sensory neuropathy (%) | 25.0 | 34.0 | 0.005 |
| Peripheral arterial disease (%) | 20.6 | 31.9 | <0.001 |
| Cerebrovascular disease (%) | 6.2 | 8.0 | 0.35 |

**Additional Table 3.** Baseline characteristics by incident heart failure (HF) status by end-June 2012 in FDS patients with type 2 diabetes and no history of HF.

|  | No HF | Incident HF | *P*-value |
| --- | --- | --- | --- |
| Number (%) | 808 (68.2) | 377 (31.8) |  |
| Age at FDS entry (years) | 61.4±11.34 | 67.5±9.7 | <0.001 |
| Sex (% male) | 48.8 | 47.2 | 0.66 |
| Ethnic background (%): |  |  | 0.34 |
| Anglo-Celt | 60.4 | 64.2 |  |
| Southern European | 17.6 | 18.3 |  |
| Other European | 8.8 | 6.9 |  |
| Asian | 4.1 | 1.9 |  |
| Indigenous Australian | 1.2 | 1.9 |  |
| Mixed/other | 7.9 | 7.7 |  |
| Not fluent in English (%) | 14.9 | 16.5 | 0.49 |
| Education beyond primary level (%) | 75.4 | 72.0 | 0.22 |
| Currently married/de facto (%) | 70.1 | 59.0 | <0.001 |
| Alcohol (standard drinks/day) | 0 [0-0.8] | 0 [0-0.8] | 0.52 |
| Smoking status (% never/ex/current) | 46.0/38.9/15.1 | 42.6/42.6/14.8 | 0.47 |
| Any exercise in past 2 weeks (%) | 76.6 | 65.8 | <0.001 |
| Age at diagnosis (years) | 56.3±11.5 | 59.8±11.2 | <0.001 |
| Duration of diabetes (years) | 3.0 [0.6-7.0] | 5.0 [2.0-11.1] | <0.001 |
| Diabetes treatment (%): |  |  | <0.001 |
| Diet | 36.3 | 23.4 |  |
| Oral agents | 54.5 | 60.4 |  |
| Insulin ± oral agents | 9.2 | 16.2 |  |
| Fasting glucose (mmol/L) | 8.4 [6.9-10.8] | 8.5 [6.9-10.9] | 0.59 |
| HbA_1c_ (%) | 7.3 [6.4-8.7] | 7.6 [6.5-8.9] | 0.022 |
| HbA_1c_ (mmol/mol) | 56 [46-72] | 60 [48-74] | 0.022 |
| BMI (kg/m^2^) | 29.5±5.7 | 29.8±5.2 | 0.36 |
| Obesity (% by waist circumference) | 61.6 | 69.6 | 0.009 |
| Systolic blood pressure (mm Hg) | 148±23 | 156±24 | <0.001 |
| Diastolic blood pressure (mm Hg) | 80±11 | 81±11 | 0.51 |
| Antihypertensive medication (%) | 42.6 | 59.2 | <0.001 |
| Angiotensin converting enzyme inhibitor (%) | 17.1 | 21.8 | 0.055 |
| Beta-blocker (%) | 14.3 | 19.1 | 0.040 |
| Calcium-channel blocker (%) | 15.0 | 27.9 | <0.001 |
| Diuretic (%) | 14.2 | 22.8 | <0.001 |
| Serum potassium (mmol/L) | 4.4±0.4 | 4.5±0.5 | 0.002 |
| Serum chloride (mmol/L) | 103±3 | 103±3 | 0.31 |
| Serum bicarbonate (mmol/L) | 27±3 | 27±3 | 0.42 |
| Total serum cholesterol (mmol/L) | 5.4±1.1 | 5.6±1.2 | 0.08 |
| Serum HDL-cholesterol (mmol/L) | 1.07±0.32 | 1.05±0.33 | 0.44 |
| Serum triglycerides (mmol/L) | 1.8 (1.1-3.2) | 2.0 (1.1-3.5) | 0.025 |
| Lipid-modifying medication (%) | 10.3 | 11.5 | 0.55 |
| Digoxin use (%) | 2.4 | 6.6 | 0.001 |
| Aspirin use (%) | 16.4 | 28.4 | <0.001 |
| Urinary albumin:creatinine (mg/mmol) | 2.3 (0.6-8.4) | 4.7 (1.0-22.1) | <0.001 |
| eGFR category (%): |  |  | <0.001 |
| ≥90 ml/min/1.73m^2^ | 26.9 | 18.7 |  |
| 60-89 ml/min/1.73m^2^ | 55.9 | 50.8 |  |
| 45-59 ml/min/1.73m^2^ | 13.4 | 19.3 |  |
| 30-44 ml/min/1.73m^2^ | 3.0 | 7.5 |  |
| <30 ml/min/1.73m^2^ | 0.9 | 3.7 |  |
| Coronary heart disease (%) | 19.1 | 37.9 | <0.001 |
| Cerebrovascular disease (%) | 6.7 | 13.0 | 0.001 |
| Peripheral arterial disease (%) | 22.3 | 39.4 | <0.001 |
| Peripheral sensory neuropathy (%) | 23.6 | 40.2 | <0.001 |
| Retinopathy (%) | 11.7 | 24.0 | <0.001 |
| Depression (%) | 28.8 | 33.5 | 0.12 |
| Antidepressant medication (%) | 6.7 | 6.4 | 0.90 |
| Schizophrenia (%) | 0.1 | 1.1 | 0.038 |
| Antipsychotic medication (%) | 1.4 | 1.3 | 1.00 |
