# Supplementary material for: Serum bicarbonate concentration and the risk of cardiovascular disease and death in type 2 diabetes: the Fremantle Diabetes Study
Source: Cardiovasc Diabetol. 2016 Oct 6;15:143. doi: 10.1186/s12933-016-0462-x (PMC5054557; doi:10.1186/s12933-016-0462-x)
Supplement: Supplementary file 2 — 10.1186/s12933-016-0462-x Most parsimonious Cox proportional hazards models (excluding serum bicarbonate) of all-cause mortality, incident coronary heart disease (CHD) and incident heart failure (HF) with age as time scale in FDS patients. [file 12933_2016_462_MOESM2_ESM.docx]

**Additional Table 4.** Most parsimonious Cox proportional hazards models (excluding serum bicarbonate) of all-cause mortality, incident coronary heart disease (CHD) and incident heart failure (HF) with age as time scale in FDS patients.

|  | All-cause mortality | | Incident CHD | | Incident HF | |
| --- | --- | --- | --- | --- | --- | --- |
|  | HR (95% CI) | *P*-value | csHR (95% CI) | *P*-value | csHR (95% CI) | *P*-value |
| Age at baseline (increase of 10 years)* | 0.32 (0.27-0.37) | <0.001 | 0.35 (0.30-0.42) | <0.001 | 0.33 (0.27-0.40) | <0.001 |
| Male | 1.59 (1.34-1.88) | <0.001 | 1.43 (1.15-1.78) | 0.001 |  |  |
| Aboriginal | 2.30 (1.22-4.35) | 0.010 |  |  |  |  |
| Not fluent in English |  |  | 0.56 (0.40-0.79) | 0.001 |  |  |
| Married/*de facto* relationship |  |  |  |  | 0.66 (0.52-0.83) | <0.001 |
| Current smoker | 1.66 (1.32-2.09) | <0.001 |  |  |  |  |
| Any exercise in the past two weeks | 0.76 (0.64-0.91) | 0.003 |  |  | 0.62 (0.49-0.78) | <0.001 |
| Diabetes duration (increase of 1 year) | 1.01 (1.002-1.03) | 0.026 | 1.03 (1.01-1.05) | <0.001 |  |  |
| Angiotensin converting enzyme inhibitor use | 1.26 (1.04-1.52) | 0.018 |  |  |  |  |
| Diuretic use | 1.22 (1.01-1.48) | 0.044 |  |  |  |  |
| Log_e_(serum triglycerides (mmol/L)) |  |  | 1.34 (1.09-1.64) | 0.005 |  |  |
| On lipid-modifying medication | 0.71 (0.54-0.94) | 0.015 |  |  |  |  |
| On digoxin |  |  |  |  | 1.72 (1.09-2.70) | 0.019 |
| Log_e_(urinary albumin:creatinine ratio (mg/mmol)) | 1.22 (1.15-1.29) | <0.001 | 1.13 (1.04-1.22) | 0.003 | 1.30 (1.21-1.40) | <0.001 |
| eGFR ≥90 ml/min/1.73 m^2^ |  |  |  |  | 1.42 (1.04-1.93) | 0.028 |
| eGFR <30 ml/min/1.73 m^2^ |  |  |  |  | 2.76 (1.45-5.27) | 0.002 |
| Retinopathy present (any grade) | 1.32 (1.06-1.65) | 0.015 |  |  | 1.63 (1.23-2.15) | 0.001 |
| Peripheral sensory neuropathy present | 1.42 (1.20-1.69) | <0.001 | 1.30 (1.02-1.66) | 0.037 | 1.68 (1.33-2.12) | <0.001 |
| Peripheral arterial disease present | 1.49 (1.25-1.76) | <0.001 | 1.55 (1.22-1.98) | <0.001 |  |  |
| History of CHD | 1.29 (1.07-1.55) | 0.007 | excluded |  | 2.03 (1.61-2.56) | <0.001 |
| History of cerebrovascular disease |  |  |  |  | 1.65 (1.18-2.33) | 0.004 |
| Charlson Comorbidity Index: |  |  | not considered |  | not considered |  |
| 0 (reference) | 1.00 |  |  |  |  |  |
| 1-2 | 1.61 (1.32-1.96) | <0.001 |  |  |  |  |
| ≥3 | 2.63 (1.94-3.57) | <0.001 |  |  |  |  |
| Schizophrenia |  |  |  |  | 4.37 (1.06-17.91) | 0.041 |

*Note that the relationship with baseline age is negative. With age as the time scale, this reflects survivor bias in the recruited cohort in that those in their 70s and 80s at recruitment, for example, are different to those recruited in their 50s and 60s. The older participants have survived long enough to reach their 70s and 80s. The 50 and 60 year olds may not survive that long. In addition, the hazards for CHD and HF are cause-specific since those that die during follow-up before they have the event of interest (CHD or HF) are censored, so the competing risk of death before the event needs to be considered when interpreting these results.
