# Supplementary material for: Serum bicarbonate concentration and the risk of cardiovascular disease and death in type 2 diabetes: the Fremantle Diabetes Study
Source: Cardiovasc Diabetol. 2016 Oct 6;15:143. doi: 10.1186/s12933-016-0462-x (PMC5054557; doi:10.1186/s12933-016-0462-x)
Supplement: Supplementary file 3 — 10.1186/s12933-016-0462-x Hazard ratios and cause-specific hazard ratios (95% CI) of serum bicarbonate concentration as quintiles and as a continuous variable for all-cause mortality, incident coronary heart disease and incident heart failure in patients with type 2 diabetes with age as time scale. (A) unadjusted; (B) adjusted for age and sex; (C) adjusted for (B) plus Aboriginal, current smoking status, any exercise in the past two weeks, not fluent in English, currently married/de facto relationship, BMI; (D) adjusted for (C) plus systolic and diastolic blood pressures, HbA1c, total and HDL-cholesterol, ln(serum triglycerides); and (E) adjusted for (D) plus ln(urinary albumin:creatinine ratio), eGFR (CKD-EPI) ≥90 or <30 ml/min/1.73m2, retinopathy, peripheral sensory neuropathy, coronary heart disease and cerebrovascular disease. [file 12933_2016_462_MOESM3_ESM.docx]

**Additional Table 5.** Hazard ratios and cause-specific hazard ratios (95% CI) of serum bicarbonate concentration as quintiles and as a continuous variable for all-cause mortality, incident coronary heart disease and incident heart failure in patients with type 2 diabetes with age as time scale. (A) unadjusted; (B) adjusted for age and sex; (C) adjusted for (B) plus Aboriginal, current smoking status, any exercise in the past two weeks, not fluent in English, currently married/*de facto* relationship, BMI; (D) adjusted for (C) plus systolic and diastolic blood pressures, HbA_1c_, total and HDL-cholesterol, ln(serum triglycerides); and (E) adjusted for (D) plus ln(urinary albumin:creatinine ratio), eGFR (CKD-EPI) ≥90 or <30 ml/min/1.73m^2^, retinopathy, peripheral sensory neuropathy, coronary heart disease and cerebrovascular disease.

|  | Quintile of serum  bicarbonate (mmol/L) | Q 1  ≤23 | Q2  24-25 | Q3  26-27 | Q4  28 | Q5  ≥29 | All  Increase of 1 |
| --- | --- | --- | --- | --- | --- | --- | --- |
| All-cause mortality | (A) | **1.27**  **(1.004-1.60)** | 1.07  (0.86-1.33) | 1.02  (0.84-1.24) | 1.01  (0.80-1.29) | 1.00  (reference) | 0.98  (0.96-1.004) |
|  | (B) | 1.20  (0.95-1.52) | 1.09  (0.87-1.36) | 0.93  (0.77-1.14) | 0.88  (0.69-1.12) | 1.00  (reference) | 0.98  (0.96-1.01) |
|  | (C) | 1.13  (0.88-1.43) | 1.02  (0.81-1.29) | 0.95  (0.78-1.16) | 0.89  (0.69-1.13) | 1.00  (reference) | 0.99  (0.96-1.02) |
|  | (D) | 1.06  (0.83-1.36) | 1.01  (0.80-1.27) | 0.92  (0.75-1.13) | 0.90  (0.70-1.14) | 1.00  (reference) | 1.00  (0.97-1.03) |
|  | (E) | 1.11  (0.85-1.46) | 0.96  (0.74-1.24) | 0.93  (0.75-1.15) | 0.99  (0.76-1.29) | 1.00  (reference) | 0.99  (0.97-1.02) |
| Coronary heart disease | (A) | **1.58**  **(1.14-2.20)** | **1.53**  **(1.12-2.10)** | 1.14  (0.86-1.53) | 0.97  (0.68-1.37) | 1.00  (reference) | **0.94**  **(0.91-0.97)** |
|  | (B) | 1.38  (0.99-1.92) | **1.39**  **(1.01-1.90)** | 0.97  (0.72-1.30) | 0.83  (0.58-1.17) | 1.00  (reference) | **0.95**  **(0.92-0.98)** |
|  | (C) | 1.34  (0.95-1.88) | 1.38  (0.99-1.91) | 0.97  (0.72-1.31) | 0.85  (0.59-1.20) | 1.00  (reference) | **0.95**  **(0.92-0.99)** |
|  | (D) | 1.29  (0.91-1.83) | 1.34  (0.96-1.87) | 0.92  (0.68-1.25) | 0.85  (0.59-1.22) | 1.00  (reference) | **0.96**  **(0.92-0.99)** |
|  | (E) | 1.23  (0.84-1.81) | 1.23  (0.86-1.75) | 0.88  (0.64-1.22) | 0.90  (0.62-1.30) | 1.00  (reference) | 0.96  (0.92-1.001) |
| Heart failure | (A) | **1.54**  **(1.13-2.09)** | 1.04  (0.76-1.43) | 1.05  (0.80-1.38) | 0.81  (0.57-1.15) | 1.00  (reference) | **0.95**  **(0.92-0.98)** |
|  | (B) | **1.41**  **(1.03-1.91)** | 0.99  (0.72-1.37) | 0.92  (0.70-1.21) | 0.72  (0.51-1.03) | 1.00  (reference) | **0.95**  **(0.92-0.99)** |
|  | (C) | 1.27  (0.93-1.75) | 0.87  (0.62-1.21) | 0.88  (0.66-1.17) | 0.70  (0.49-1.17) | 1.00  (reference) | 0.97  (0.93-1.003) |
|  | (D) | 1.14  (0.82-1.57) | 0.84  (0.60-1.18) | 0.85  (0.63-1.13) | 0.70  (0.49-1.01) | 1.00  (reference) | 0.98  (0.94-1.02) |
|  | (E) | 1.08  (0.76-1.55) | 0.78  (0.54-1.13) | 0.87  (0.64-1.18) | 0.71  (0.48-1.05) | 1.00  (reference) | 0.99  (0.95-1.03) |
